# Human Breast Milk: Exploring the Linking Ring Among Emerging Components

**DOI:** 10.3389/fped.2018.00215

**Published:** 2018-08-07

**Authors:** Flaminia Bardanzellu, Vassilios Fanos, Francesca A. L. Strigini, Paolo G. Artini, Diego G. Peroni

**Affiliations:** ^1^Neonatal Intensive Care Unit, Neonatal Pathology and Neonatal Section, Azienda Ospedaliera Universitaria di Cagliari, University of Cagliari, Cagliari, Italy; ^2^Gynecology and Obstetrics, Università degli Studi di Pisa, Pisa, Italy; ^3^Section of Pediatric, Department of Clinical and Experimental Medicine, University of Pisa, Pisa, Italy

**Keywords:** human milk, metabolomics, microbiota, microbiomics, human milk oligosaccharides, preterm, newborn

## Abstract

Maternal breast milk (BM) is a complex and unique fluid that evolution adapted to satisfy neonatal needs; in addition to classical nutrients, it contains several bioactive components. BM characteristically shows inter-individual variability, modifying its composition during different phases of lactation. BM composition, determining important consequences on neonatal gut colonization, influences both short and long-term development. Maternal milk can also shape neonatal microbiota, through its glycobiome rich in *Lactobacilli* spp. and *Bifidobacteria* spp. Therefore, neonatal nourishment during the first months of life seems the most important determinant of individual's outcomes. Our manuscript aims to provide new evidence in the characterization of BM metabolome and microbiome, and its comparison to formula milk, allowing the evaluation of each nutrient's influence on neonatal metabolism. This result very interesting since potentially offers an innovative approach to investigate the complex relationship between BM components and infant's health, also providing the chance to intervene in a sartorial way on diet composition, according to the nutritional requests. Future research, integrating metabolomics, microbiomics and stem cells knowledge, could make significant steps forward in understanding BM extraordinary properties and functions.

## Introduction

Breast Milk (BM) is a precious fluid which has been considered miraculous since ancient times. Its extraordinary properties have been studied in detail, not resulting fully clarified yet. It can confer protection against a large number of pathologies and exerts a beneficial effect on breastfed newborn's development (1, 2).

BM is the most suitable choice for neonatal nutrition, highly recommended as the exclusive component of the infant's diet for almost 6 months of life (3).

Nutrition in the early neonatal period influences the successive whole life, due to its role in the activation of several metabolic processes, for example, microanatomy development, growth, metabolism, gut microbiological colonization and maturation, immunological system development, brain maturation, and organization (1, 4).

In fact, BM has been associated to many beneficial short-term effects, such as a reduction in necrotizing enterocolitis (NEC) and sepsis (5); a positive influence on long-term outcome (such as neurodevelopment) and a protective effect against infections, overweight, obesity, diabetes and malignant diseases incidence have also been described (6).

BM beneficial effects do not regard exclusively infants' health but could also be exerted on lactating mothers, improving their outcome (6).

BM contains several components, such as lipids, carbohydrates, proteins, vitamins, minerals. Oligosaccharides, the third most abundant constituents of BM, which represent a highly variable fraction of BM, exert several important functions, such as the modulation of neonatal gut microbiota composition, influencing many physiological processes (7).

BM is also defined an “alive” fluid, since it provides to the breastfed newborn maternal soluble bioactive components, growth-factors (GFs), hormones, cytokines, chemokines, immunoglobulins (Ig), and immunological-related cells as leucocytes, cells of both bacterial and maternal origin and finally, as recently demonstrated, also multipotent Stem Cells (SCs) able to integrate *in vivo* in many neonatal tissues and differentiate into mature cells. Finally, great relevance can also be attributed to the presence of maternal milk microbiota (1, 2, 5, 7–9).

BM composition has the extraordinary property to vary according to gestational age (GA) of the neonate and to the lactation phase (5, 6, 10).

Since the degree of prematurity highly influences BM features, the resulting composition is optimal for preterm newborns needs. Macro- and micronutrients levels vary and determine advantages regarding immunity, neurological development, gastrointestinal maturation (9, 11–17).

The presence of several cytokines and chemokines, showing a higher concentration in colostrum, has been widely evidenced and could represent an additional mechanism of protection, especially against NEC and sepsis (17, 18).

It is not fully known how maternal or pregnancy factors could modify their level, although in peripartum infections, in spontaneous preterm delivery and in VLBW neonates lower concentrations of pro-inflammatory cytokines have been measured, potentially protecting against mucosal damage or pathogens (19).

Even the cellular composition of BM varies among samples deriving from mothers of preterm and full-term newborns, meeting the necessities showed by premature neonates during the first phases of life (9) and confirming the extraordinary ability of this liquid to modify itself according to the newborns features and assuming the best qualities for his optimal development (5).

BM related SCs belong to different lineages such as mammary epithelial cells, neuroepitelial-like SCs and mesenchimal SCs (10–15%) (20).

It seems that, among large amount of SCs ingested by the newborns each day, some can pass from BM through neonatal gut and migrate into brain and other organs; there, they can persist and proliferate as in a microchimerism, restoring the involved organs, potentially even after a damage (1, 5, 21). This interaction between the dyad mother-child result very interesting but all the implications should be deeper clarified.

## Metabolomics

The great relevance of micronutrients in BM is highlighted by the increasing number of metabolomic studies performed to characterize its metabolic profile and inter- and intra- individual variations (8, 10, 16, 22–30).

BM can be analyzed through nuclear magnetic resonance (NMR) and liquid or gas chromatography coupled with mass spectrometry (LC-MS or GC-MS), to evaluate its unique profile (5).

The first metabolomic study investigating BM composition was conducted by Cesare Marincola et al in 2012 (22). This group demonstrated different metabolic features characterizing subsequent lactation stages. Moreover, they found higher levels of lactose and lower levels of maltose in BM samples, compared to formula milk (FM) (1, 17, 22).

Interesting results were also obtained by the numerous successive studies, performed by several groups. The most relevant findings are reported below, and they can be found in a more detailed way and summarized in 2 tables in the recent papers published by Fanos et al. (5) and Bardanzellu et al. (17).

According to the findings of Spevacek and colleagues, (23) the highest variability can be found in preterm samples. They identified and measured variations in 69 metabolites and also demonstrated that lacto-N-tetraose and lysine decreased during the maturation of full-term milk (23).

Another group (10) demonstrated that preterm BM metabolome mostly varies within 5–7 weeks postpartum; after this period it would probably obtain the composition of term milk after this time and BM dependence on GA seems to be reduced (5, 10, 17, 31).

Moreover, in colostrum samples from preterm delivering mothers, an increased level of fucosylated oligosaccharides, fucose, N-acetylneuramic acid and N-acetylglucosamine, citrate and creatinine have been shown (10).

The group of Villasenor et al. also demonstrated a different composition between full term colostrum and mature milk and moreover, our research group detected a higher sample variability in colostrum instead of mature milk belonging to extremely preterms (27).

Summarizing these findings, the highest variability has been evidenced during the first three months of lactation (25), with a high dependence on GA (28); a different metabolitic pattern comparing human colostrum with transition milk and mature was observed (23).

BM from mothers of preterm neonates showed a different composition if compared with full-term newborn mothers' BM. In particular, a higher concentration of proteins and aminoacids, promoting cerebral development and energy production, was observed. This reinforces the concept of BM variability according to the breastfed newborn's peculiarities (17).

Among the genetic factors highly influencing BM composition, four maternal phenotypes, depending on both blood group and expression of two specific genes, were identified.

This influence in particular regards human milk oligosaccharides (HMOs), which constitute the third most abundant solid fraction of BM, following lactose and lipids (1.9–4.5%) (7).

These genes are, firstly, alpha-1-2-fucosyltransferase (secretor gene, FUT2) which is codified by *Se* gene and allows the classification of secretors (*Se*^+^) and non-secretors (*Se*^−^) mothers. Secondly, it is considered alpha-1-3-4-fucosyltransferase gene(Lewis gene, FUT3); it indicates positivity or negativity for Lewis Group (*Le*^+^ or *Le*^−^). According to these considerations, maternal phenotypes can be divided in: *Se*^+^*/Le*^+^*, Se*^+^*/Le*^−^*, Se*^−^*/Le*^+^, and *Se*-/Le^−^, showing significant differences in BM metabolites (1, 17, 32, 33).

In fact, *Se*^+^*/Le*^+^ mother's BM exhibits all the fucosylated oligosaccharides (2′fucosyl-lactose 2′FL, lactodifucotetraose LDFT, Lacto-N-fucopentaose I LNFPI, Lactodifucoesaose I LNDFHI), while the *Se*^−^*/Le*^+^ mother's phenotype determines the production of samples containing a high concentration of HMOs with (α1-3) and (α1-4)-linked fucose residue, in absence of α1,2-fucosylated structures (32, 34–36).

The great interest concerning BM HMOs composition is also related to their potential influence on microbiota (10, 37, 38).

It has been estimated that FUT2 is expressed in more than 70% of the Caucasian women (34). The absence of α1-2-fucosylated oligosaccharides in BM can lead to several pathophysiological consequences, such as a delayed colonization by *Bifidobacteria* spp., a higher abundance of *Streptococcus* spp. and also functional differences of microbiota metabolic activity. According to some authors, *Se*^+^*/Le*^+^ phenotype results protective against some infections, such as *E. coli* and *Campylobacter* spp. and preventive of NEC (35), while infants fed with BM from *Se*^−^mothers would show a higher risk for diarrheal diseases (11, 39, 40).

In accordance with these data, Bazanella and colleagues analyzed BM samples from *Se*^+^ mothers, demonstrating a higher percentage of fucosylated oligosaccharides instead of *Se*^−^ mothers, and the presence of *B. longum* exclusively in the stools of *Se*^+^ breastfed neonates (41).

HMOs are known to decrease with milk maturation (10, 27). According to many studies, total HMOs content, sialic acid, lacto-N-tetraose, LNDFH I, 3′- sialyllactose, 6′-siallylactose, fucose, N-acetylglucosamine, N-acetylneuraminic acid resulted higher in preterm milk (10, 23, 42).

In addition to HMO's, also amino acids and lipids showed a great variability across lactation stages and a great dependence on prematurity. Some amino acids increase, while other reduce their concentration during BM maturation (10, 17, 43).

The studies performed in this field allow to conclude that BM, in particular in the first phases and in the samples obtained by premature delivering mothers, is extremely rich in creatinine and amino acids. These factors are involved in two crucial processes, especially for the vulnerable category of preterm babies: brain development and energetic metabolism (17).

In particular, creatinine, betaine, coline, leucine, isoleucine, and valine take part in cerebral maturation (10, 26, 28, 44, 45), while energy production is closely related to the presence of alanine, glutamate, methionine and creatinine (23, 26, 28, 46, 47).

Coline and betaine could play a role in the reduction of cardiovascular diseases (28, 48). Moreover, acetylcarnitine, betaine, lysine, isoleucine, and taurine levels seem to decrease during milk maturation in samples of mother of full term neonates and not in preterm samples (17).

Other detected metabolites may also take part in several immunity processes, hepatic regeneration, lipid and glucidic metabolism (17, 45, 49–51).

It is also been demonstrated that fatty acids' (FA) composition in BM can be influenced by many factors, not fully understood up to now. Among these, maternal age, nationality, parity, body mass index (BMI), diet, newborn's GA, lactation stage, maternal gestational diabetes mellitus, number and duration of breastfed meals and delivery route can be mentioned, although the entity of their influence is not currently attested. The most represented fractions are tryglicerides, palmitic, oleic, linoleic and alpha-linolenic acids (17, 52–60).

FA's content seems to be higher in colostrum from mothers whose neonates' weight was lower than the 20° centile (52, 61, 62) and this mechanism may probably help to compensate the intrauterine growth restriction occurred in these neonates.

According to the analysis of Collado et al. (63), evaluating BM from preterms and full-term delivering mothers, the content of FAs resulted comparable among colostrum and mature milk samples, although different qualitative profiles were found.

Recently, interesting results confirmed the importance of a metabolomic approach to evaluate the differences occurring in newborns fed with BM or formula milk during the early life. Cesare Marincola et al. (64) detected variable urinary profiles in relation to the kind of diet; several and more numerous trials would be needed to fully understand the clinical implications of these findings, improving our knowledge on BM's effects.

Metabolomics also gave promising results analyzing urine and/or blood samples of breastfed neonates or those from their mothers, to understand biological effects of maternal BM. Two recent studies evidenced as different metabolites can be found in urine or blood of breastfed newborns, instead of those described in samples belonging to FM fed newborns (31, 65).

Moreover, the analysis of urine from breastfeeding mothers revealed different profiles too (5, 66).

In conclusion, BM metabolome varies according to GA and lactation phase, depending on neonatal necessities and especially meeting the peculiar requests of the vulnerable category of premature newborns.

## Microbiomics

Even if BM was considered sterile for long time, it has recently been demonstrated, through culture-dependent and -independent techniques, that the microbial community in BM from healthy mothers can contain more than 200 phylotypes, belonging to about 50 different genera (2, 67, 68).

The technological advances, particularly the cultivation independent methods, such as 16S gene sequencing, allowed a deeper analysis of bacterial diversity, giving more detailed information on the populations present in several human fluids, such as BM.

In this sampling, although they permitted to demonstrate a bacterial load between two and three orders of magnitude, which resulted higher than those estimated by cultures, from a critical point of view these technique are not able to discriminate DNA sequences from non-vital bacteria and extracellular DNA that could interfere the amplification by quantitative PCR (qPCR). However, it is also clear that the early stimulations coming from all the microbial products, alive or residual, could influence the newborn immune system (69).

According to the current knowledge, how maternal microbes can reach mammary epithelium and undergo secretion in BM, is still matter of debate. Milk harbored bacteria may derive from the contamination with bacteria from mother's skin (such as *S. epidermidis*) and from infant's oral cavity. On the other side it has been postulated that microorganisms from maternal intestinal tract can reach the mammary gland through a vascular transport via intestinal immune cells, especially dendritic cells (the so-called entero-mammary pathway hypothesis). This entero-mammary pathway allows to consider as maternal gastrointestinal bacteria during pregnancy and lactation could directly influence the infant's immune system. Moreover, a retrograde flow of newborn's microbes could occur during nursing (17, 67, 68, 70, 71). However, due to its peculiar characterization, this community is even more considered as a site-specific microbiota, as demonstrated by several anaerobic species that are identifiable and that are not present both in the skin or in the oral cavity (2, 67, 68).

This community is represented, for about half, by a constant core microbiota with a limited variability, being BM microbiota dominated by *Staphylococcus* spp.*, Pseudomonas* spp., *Streptococcus* spp., *Acinetobacter* spp.*, Finegoldia* spp., *Anaerococcus* spp., *Actinomyces* spp., and *Enterobacter* spp. (67, 69), with huge differences between colostrum and mature milk (68, 69, 72–77).

On the contrary, the other half seems to be highly dependent on maternal factors, such as ethnicity, diet, drug exposure, environmental factor exposure, mode of delivery (67, 69).

BM is an exceptional source of commensal bacteria for breastfed newborns, representing a dynamic ecosystem for several species, which can modify itself during milk maturation, according to the infant's needs (67, 72). In fact, microbiota shows high variability during the subsequent lactation stages, also highlighted by the possibility to detect different microbial-related metabolites (69, 78). After about one month of lactation, BM microbiota reaches the full maturation and its definitive composition, maintaining therefore a relative stability (67).

Composition and metabolic network of BM microbiota may be considered as an epigenetic determinant of neonatal health (78, 79). Moreover, many microbic-related metabolites represent a linking ring between metabolomics and microbiomics. In particular, BM microbiota is known to shape the newborn intestinal microbiota since the early phases of life. Bacterial genera and species present in colostrum and then in mature milk can positively influence intestinal bacterial network, highly rich in *Bifidobacteria* spp. and *Lactobacilli* spp. Bacterial communities produce metabolites, such as short chain fatty acids (SCFA), mostly butyrate, that may be detected in the fluids by metabolomics and are able to influence several health outcomes of the child.

Moreover, an active role is played by sialylated BM HMOs, which can induce transcriptional responses in the intestinal microbiota (i.e., *B. Fragilis*) potentially influencing even other microbial members, such as *E. coli*. Therefore, through several routes and deep interactions not fully clarified yet, this leads to infant growth promotion, beneficial metabolic pathways and effects on several organs (brain, liver, respiratory, and urinary tract) (36, 80).

The effect of early diet on pigs' intestinal microbiota has been also evaluated by Piccolo et al. (81), demonstrating that neonatal nutrition could characteristically induce different effects according to the different small bowel's region, mostly influencing duodenum microbial composition, since microbial network seems to be functionally defined by the intestinal segment (81).

This bioregional effect of nutrition on the shape of intestinal microbiota also influences the production of different molecules, highlighting the strict dependence of metabolic profile from microbial communities and the influence played by microbiota itself on the host tissue metabolism, as demonstrated through metabolomic analysis (81).

Hunt et al. (68) observed as BM microbial community represents a unique fingerprint characterizing each mother's sample. BM and therefore neonatal intestinal microbiota can be influenced by several factors, as reported in Table 1, including genetics (such as secretor status, as previously described), delivery route (in relation to newborn's colonization with maternal vaginal microbiota during spontaneous delivery), maternal weight, diet and lifestyle (in relation to ingested foods and even to maternal diseases or metabolic status), antibiotic therapy administered just before delivery (influencing maternal intestinal microbes), environmental factors, lactation stage or GA (recognized as actors influencing intestinal metabolites and HMOs and thus, indirectly, newborn's microbial community), mastitis or maternal dysbiosis (allowing the newborn to become in contact with potentially dangerous microbial communities) (2, 17, 67, 72, 82, 84, 87).

**Table 1:** Factors influencing the composition of breast milk and/or neonatal intestinal microbiota and some of proposed mechanisms and exerted effects.

**Maternal factors**	**Effects on BM and/or neonatal gut microbiota**
Genetics (1, 16, 26, 27)	Secretor status *Se^+^* (associated with high presence of *Bifidobacteria* spp. in neonatal stools) and non secretor status *Se^−^* (higher percentage of *Streptococcus* spp.), Lewis gene; ethnicity; other factors not completely known.
Lactation phase (54, 57–59, 63)	Modulation of BM metabolites and microbial community, directly influencing neonatal gut microbiota and metabolic network
Breast milk composition (such as oligosaccharides, lipids) (10, 37, 38, 72)	Modulation of BM metabolites and microbial community, directly influencing neonatal gut microbiota and metabolic network. HMOs influence *B. Fragilis, E. coli etc…* HMOs and FAs influence *Bifidobacteria s*pp. and *Staphylococci* spp. in neonatal gut
Body mass index (5, 82, 83)	Influence on maternal metabolic status
Diet, lifestyle and habits (2, 5, 17, 67, 82)	Influence played by ingested foods, maternal diseases or metabolic status
Delivery route (vaginal, elective or emergency cesarean section) (5, 72, 82–85)	Induce neonatal colonization with maternal vaginal microbiota during spontaneous delivery. *S. Salivarius* detected only in BM samples from mothers undergone cesarean section. Other factors not completely known
Gestational age at delivery (86)	Modulation of BM metabolites and microbial community, directly influencing neonatal gut microbiota and metabolic network
Administration of antibiotics (17, 33)	Influence on maternal intestinal microbiota
Dysbiosis and/or mastitis (2, 17, 73, 74)	Neonatal contact with potentially dangerous microbial communities

Due to such reasons, maternal intestinal microbiota influences breastfed newborn, modulating its intestinal microbial community. In fact, several factors leading to maternal dysbiosis (such as an overgrowth of a microbial species instead of the others) and/or breast infection, which has been associated to a reduced variability in microbiota composition, can show direct effects on breastfed neonate's health (2, 73, 74).

Among the other factors, even BM lipid composition, in addition to maternal BMI, seems potentially influence BM microbiota (72, 74).

Finally, even the modality of delivery can modify qualitative bacterial composition of BM. For example, *S. Salivarius*, an oral commensal, was detected only in samples collected from mothers who underwent cesarean section (72, 85).

It is clear that BM microbiota plays a central role even in the early colonization of neonatal gastro-enteric tract (67, 88), considering that the breastfed newborn swallows about 1 x 10^5−8^ bacteria/day (67–69, 71, 72, 88, 89). Since BM contains both probiotics (such as *Bifidobacteria* spp. and *Lactobacilli* spp.) and prebiotics (mostly HMOs) it can be considered a natural symbiotic mixture (72, 75).

Thus, neonatal commensal bacteria could be involved in gut tolerance modulation, immune system stimulation and may even inhibit reactions vs. some DNA fragments (78, 90, 91). This would result in a greater protection against several diseases, reducing the rate of enteric and respiratory infections (67, 71, 92).

Moreover, in BM, there are many anaerobic and lactic acid bacteria (69), which could confer further anti-microbial protection and improve nutrients' absorption (67, 68, 71, 72, 88–91, 93).

The recent study of Damaceno et al. (72), investigating BM microbiota in healthy mothers, revealed a bacterial concentration ranging from 1.5 to 4.0 log_10_ CFU/mL, with the highest concentration in colostrum. In their sample, *S. epidermidis* resulted the predominant species.

Our group (67) evaluated microbiota network in italian mothers, detecting a variable microbial composition duringprogressive lactation stages and even some differences occurring among different populations. In particular, colostrum of Italian mothers mostly contained *Abiotrophia* spp., *Actynomicetospora* spp.*, Aerococcus* spp.*, Alloicoccus* spp., *Amaricoccus* spp.*, Bergeyella* spp.*, Citrobacter* spp.*, Desulfovibrio* spp.*, Dolosigranulum* spp.*, Faecalibacterium* spp.*, Parasutterella* spp.*, Rhodanobacter* spp.*, Rubellimicrobium* spp. (67, 88, 94). Other authors also demonstrated a high prevalence of *Weisella* spp.*, Leuconostoc* spp.*, Staphylococci* spp.*, Streptococci* spp., and *Lactobacilli* spp. (82).

In mature BM from italian mothers, *Abiotrophia* spp. and *Aerococcus* spp. were also present, in addition to *Acetanaerobacterium* spp., *Aciditerrimonas* spp., *Acidocella* spp., *Aminobacter* spp., *Bacillus* spp., *Caryophanon* spp., *Delftia* spp., *Microvirga* spp., *Parabacteroides* spp., *Phascolarctobacterium* spp., and *Alistipes* spp. (67). Other authors also reported the presence of *Veillonella* spp., *Leptotrichia* spp., *Prevotella* spp. (82)., *Enterococcus* spp., *Lactococcus* spp., *Actinomyces* spp., *Corynebacterium* spp., *Kecuria* spp., *Escherichia coli* spp., *Klebsiella* spp., and *Raistonia* spp. species (88).

In the same study evaluations were conducted in colostrum and mature milk from mothers living in Burundi, where so many genetic but also environmental factors can be taken into account to explain huge differences in microbial composition. Several differences in the microbiota network have been observed also in different lactation stages of the same population and it is clear that the differences between these two populations may influence the findings (67).

Analyzing the gut microbiota of breastfed neonates, and comparing it to FM fed infants, different levels of *Proteobacteria* spp., *Bacteroides* spp., *Actinobacteria* spp., and *Firmicutes* spp. were detected (95); moreover, *Bifidobacteria* spp. resulted one of the most represented species, especially *Bifidobacterium longum* subsp. *longum* and *infantis*, and *B. breve* (96), which also showed a high concentration in breastfed neonate's stools (2, 86).

*Bifidobacteria* spp., *Lactobacilli* spp., *and Bacteroides* spp. proliferation is useful to face intestinal aggressive pathogens' invasion (such as *Salmonella* spp., *Lysteria* spp., and *Campilobacter* spp. (97, 98). Moreover, BM has a buffering capacity, that allows acidifying the intestinal content in order to make it more fermentable by the bacteria of the proximal colon. BM shows an inhibitory effect on the growth of *Clostridi* spp., *Bacteriodes* spp. and other anaerobic bacteria.

The great influence exerted by BM on neonatal intestinal microbial composition allow to indicate this community with the expression milk-oriented microbiota (MOM) (1).

This effect mainly occurs through the action of the glycans, constituted by free HMOs, glycolipids and glycoproteins and highly contained in BM. In fact, as previously reported, they act as prebiotics, since they do not undergo absorption in proximal gut (38, 99) and represent growth substrates for specific hugs (67, 68, 71, 72, 88, 96, 100–103).

Therefore HMOs, and even FAs, influence the growth of some beneficial species in neonatal gut, such as *Bifidobacteria* spp. and *Staphylococci* spp., related to several positive effects (38, 96, 104–106). For example, a better response to vaccines, an improved function of the intestinal barrier and a protection against intestinal infections (38, 107–109).

Moreover, bacterial species like *Bacteroides* spp., *Bifidobacteria* spp. and *Lactobacilli* spp. are very important for HMOs' metabolism (72), promoting their degradation into sugars available for energy production. In addition, *Bifidobacteria* spp.*, Lactobacilli* spp., *and Bacteroides* spp. can induce short chain fatty acids (SCFAs) production, playing a role in gut mucosa homeostasis and in lipid metabolism (97, 98).

A recent study of Karav et al. (96) demonstrated that a endo-β-N-acetylglucosaminidase (EndoBI-1) found in several *Bifidobacteria* spp. promotes the cleavage of N-linked glycans fragments. These can influence bacterial selective growth, especially allowing *Bifidobacterium longum* subsp*. Infantis* (*B. infantis*) proliferation and even interfering with other subspecies' metabolism. On the contrary, in the same study, *B. infantis* did not result able to grow exclusively in presence of the de-glycosylated protein fraction, confirming the role played by glycans in bacterial growth.

In this perspective, *Bifidobacteria* spp. and other species able to perform the initial de-glycosylation seem advantaged, since this represents a key passage. In fact, many studies demonstrated that the shape of gut microbiota, through the influence of microbial growth, is better promoted by HMOs and deconjugated glycans instead of the whole glycoprotein or glycolipid (96, 110–120).

This topic represents an interesting field of research, being not full clarified up to now. It would be very promising to find a link among the exact and inter-individual BM composition, the exerted influence on intestinal microbiota of the newborn and its resulting clinical phenotype. In fact it could be a suitable substrate for therapeutic beneficial applications modifying the final outcomes.

For example, BM content in HMOs and even neonatal gut microbiota showed some differences in under nutrition models, leading to an impaired infantile development. Therefore, it could be very useful to perform some dietetic strategies, adapted to these needs, which could treat or prevents several disorders, including under nutrition (38, 121).

## Conclusions

BM exceptional features make it a very precious fluid, whose extraordinary properties and functions have not been fully clarified yet. It would be very interesting to understand all maternal factors influencing BM composition, also regarding SCs, in terms of quality and quantity (21, 122).

In the last years the importance of metabolomics has been highlighted, especially due to its role in characterizing metabolites related to microbial network. This integrated approach to the triad nutrients-microbes-metabolites can allow the identification of the effective bacterial taxa in BM and therefore transferred to the newborn (78, 123, 124), since we know that BM is the best modulator of neonatal microbiota (125). These findings would help to clarify, and even predict, BM influence on neonatal short- and long-term outcomes. Moreover, such observations may result useful to perform a sartorial approach through targeted strategies which potentially could, improve neonatal or even maternal health through the modulation of BM microbiota (2).

Finally, these evidences suggest the possible importance of bacterial supplementation of FM. The detailed knowledge of BM composition could allow to produce the best artificial products to provide to the nourished newborn a FM resembling, in the most accurate way, BM characteristics (78, 126).

## Author's note

DP is affiliated with the International Inflammation Network (in-FLAME) of the World Universities Network.

## Author contributions

FB made the selection of the papers from the literature. FB wrote the paper. VF and DP conceived the paper and FS and PA revisioned the manuscript.

### Conflict of interest statement

The authors declare that the research was conducted in the absence of any commercial or financial relationships that could be construed as a potential conflict of interest. The reviewer LA and handling Editor declared their shared affiliation.
